# Ancestral Genomic Functional Differences in Oligodendroglia: Implications for Alzheimer’s Disease

**DOI:** 10.21203/rs.3.rs-5338140/v1

**Published:** 2024-12-04

**Authors:** Aura M Ramirez, Luciana Bertholim-Nasciben, Sofia Moura, Lauren E Coombs, Farid Rajabli, Brooke A. DeRosa, Patrice G Whitehead, Larry D Adams, Takiyah D Starks, Pedro Mena, Maryenela Illannes-Manrique, Sergio J Tejada, Goldie S Byrd, Allison Caban-Holt, Michael Cuccaro, Katalina McInerney, Mario Cornejo-Olivas, Briseida Feliciano-Astacio, Liyong Wang, Maria C Robayo, Wanying Xu, Fulai Jin, Margaret A Pericak-Vance, Anthony J Griswold, Derek M Dykxhoorn, Juan I Young, Jeffery M Vance

**Affiliations:** University of Miami Miller School of Medicine: University of Miami School of Medicine; University of Miami Miller School of Medicine: University of Miami School of Medicine; University of Miami Miller School of Medicine: University of Miami School of Medicine; University of Miami Miller School of Medicine: University of Miami School of Medicine; University of Miami Miller School of Medicine: University of Miami School of Medicine; University of Miami Miller School of Medicine: University of Miami School of Medicine; University of Miami Miller School of Medicine: University of Miami School of Medicine; University of Miami Miller School of Medicine: University of Miami School of Medicine; Wake Forest School of Medicine: Wake Forest University School of Medicine; University of Miami Miller School of Medicine: University of Miami School of Medicine; Global Brain Health Institute; University of Miami Miller School of Medicine: University of Miami School of Medicine; Wake Forest School of Medicine: Wake Forest University School of Medicine; Wake Forest School of Medicine: Wake Forest University School of Medicine; University of Miami Miller School of Medicine: University of Miami School of Medicine; University of Miami Miller School of Medicine: University of Miami School of Medicine; Universidad Científica del Sur Facultad de Ciencias de la Salud: Universidad Cientifica del Sur Facultad de Ciencias de la Salud; Universidad Central Del Caribe Escuela De Medicina: Universidad Central del Caribe School of Medicine; University of Miami Miller School of Medicine: University of Miami School of Medicine; University of Miami Miller School of Medicine: University of Miami School of Medicine; Case Western Reserve University School of Medicine; Case Western Reserve University School of Medicine; University of Miami Miller School of Medicine: University of Miami School of Medicine; University of Miami Miller School of Medicine: University of Miami School of Medicine; University of Miami Miller School of Medicine: University of Miami School of Medicine; University of Miami Miller School of Medicine: University of Miami School of Medicine; University of Miami Miller School of Medicine: University of Miami School of Medicine

## Abstract

**Background:**

This study aims to elucidate ancestry-specific changes to the genomic regulatory architecture in induced pluripotent stem cell (iPSC)-derived oligodendroglia, focusing on their implications for Alzheimer’s disease (AD). This work addresses the lack of diversity in previous iPSC studies by including ancestries that contribute to African American (European/African) and Hispanic/Latino populations (Amerindian/African/European).

**Methods:**

We generated 12 iPSC lines—four African, four Amerindian, and four European— from both AD patients and non-cognitively impaired individuals, with varying *APOE* genotypes (*APOE3/3* and *APOE4/4*). These lines were differentiated into neural spheroids containing oligodendrocyte lineage cells. Single-nuclei RNA sequencing and ATAC sequencing were employed to analyze transcriptional and chromatin accessibility profiles, respectively. Differential gene expression, chromatin accessibility, and Hi-C analyses were conducted, followed by pathway analysis to interpret the results.

**Results:**

We identified ancestry-specific differences in gene expression and chromatin accessibility. Notably, numerous AD GWAS-associated genes were differentially expressed across ancestries. The largest number of differentially expressed genes (DEGs) were found in European vs. Amerindian and African vs. Amerindian iPSC-derived oligodendrocyte progenitor cells (OPCs). Pathway analysis of *APOE4/4* carriers vs *APOE3/3* carriers exhibited upregulation of a large number of disease and metabolic pathways in *APOE4/4* individuals of all ancestries. Of particular interest was that *APOE4/4* carriers had significantly upregulated cholesterol biosynthesis genes relative to *APOE3/3* individuals across all ancestries, strongest in iOPCs. Comparison of iOPC and iOL transcriptome data with corresponding human frontal cortex data demonstrated a high correlation (R^2^ > 0.85).

**Conclusions:**

This research emphasizes the importance of including diverse ancestries in AD research to uncover critical gene expression differences between populations and ancestries that may influence disease susceptibility and therapeutic interventions. The upregulation of cholesterol biosynthesis genes in *APOE4/4* carriers of all three ancestries supports the concept that *APOE4* may produce disease effects early in life, which could have therapeutic implications as we move forward towards specific therapy for *APOE4* carriers. These findings and the high correlation between brain and iPSC-derived OPC and OL transcriptomes support the relevance of this approach as a model for disease study.

## BACKGROUND

Alzheimer’s disease (AD) is a devastating neurodegenerative disorder characterized by progressive cognitive decline and memory loss. Approximately 11% of people aged ≥ 65 years have AD and this percentage increases with age, reaching 13% of people aged 75–84 years and 33% of people aged 85 years or older. Although age is the strongest risk factor for AD development, genetics plays a critical role in driving AD risk. To date, more than 100 genetic loci have been recognized as predisposing factors for AD through genome-wide association studies (GWAS)[1–8]. Most of these studies have focused on data acquired from non-Hispanic white individuals, with African, African American, and Hispanic/Latino populations being largely underrepresented [9]. Recently, expanding the representation of these populations in genetic studies has been a growing area of focus, driven in large part by large multicenter collaborations, such as the Alzheimer’s Disease Sequencing Project (ADSP) [10]. Both Hispanic/Latino and African American populations are admixed populations. Hispanic/Latino individuals’ have differing combinations of European, Amerindian, and African ancestry, while African Americans have primarily African and European ancestry. As such, expanding the representation of other genomic compositions in genomic studies is imperative, as there is clear evidence of differences in risk loci and effect sizes of risk alleles shared across populations. For example, the ε4 allele of the apolipoprotein E (APOE) gene represents the most prominent genetic susceptibility factor for late-onset AD, exerting the most pronounced effect on East Asian (EA) populations (Japanese), followed by non-Hispanic White (European) populations, with a comparatively smaller effect in populations of African descent [11–13]. The mechanisms underlying these differences have been shown to be due to ancestry-specific variations in the genomic regulatory architecture (GRA) of the local ancestry surrounding APOE [14–16]. Notably, GRA is not only ancestry biased but also cell type specific.

While extensive research has focused on the role of neurons in AD pathogenesis, recent evidence suggests that glial cells, including oligodendrocytes, play crucial roles in the etiology of AD. Oligodendrocytes, which are traditionally known for their role in myelinating axons in the central nervous system [17], have emerged as key players in AD due to their involvement in various AD-associated pathological processes, including neuroinflammation, oxidative stress, and synaptic dysfunction [18]. For example, many AD risk genes such as *APP, ANK3, BACE1, BIN1, PICALM, PSEN1*, and *SORT1* are highly expressed in oligodendrocytes. Additionally, phosphorylated tau in gray matter has been associated with white matter abnormalities and demyelination in AD patients, with these abnormalities appearing prior to the onset of clinical AD symptoms [19], suggesting the importance of oligodendroglia in AD pathogenesis. For these reasons, we examined and compared the GRA of iPSC-derived oligodendroglia from individuals with significant African (AF), Amerindian (AI), or European (EU) global ancestry. Taken together, our results provide a resource for the AD research community to gain knowledge of genetic factors that may be specific to certain populations, thereby providing a more comprehensive understanding of the disease.

## METHODS

### Sample collection.

Samples were obtained through genetic studies of AD in African-Americans (R01AG072547 and U01-AG052410), the Puerto Rico Alzheimer Disease Initiative (PRADI) [20, 21], and an ongoing study of AD in Peru (R01-AG070864) [12]. The study was approved by the Institutional Review Boards (IRBs) of the University of Miami and the Universidad Peruana Cayetano Heredia (Lima, Peru). All participants provided informed consent in accordance with the guidelines and regulations of the University of Miami. Cognitive data was obtained on all individuals and was reviewed by a boarded neurologist (J.M.V.), and neuropsychologists (M.C., K.M, A. C-H.) for clinical diagnosis.

### Induced pluripotent stem cells (iPSCs).

Peripheral blood mononuclear cells (PBMCs) were extracted from whole blood using SepMate^™^−50 tubes with Lymphoprep^™^ media by density gradient centrifugation according to the manufacturer’s protocol (STEMCELL Technologies). PBMCs from AD patients or aged-matched nondemented controls were selected based on their global ancestry (> 90% European, African or Amerindian) (Table 1) to be reprogrammed using the CTS^™^ CytoTune^™^-iPS 2.1 Sendai Reprogramming Kit (Invitrogen) to generate iPSCs in the Hussman Institute for Human Genomics iPSC Core Facility.

### Global ancestry estimation

The admixture proportion was calculated using a model-based clustering algorithm within ADMIXTURE software [22]. A supervised ADMIXTURE analysis was conducted at K = 4, incorporating the 4 reference populations (AI, EU, AF, and EA) from combined reference panels of the Human Genome Diversity Project (HGDP) [23, 24] and 1000 Genomes Phase 3 [25, 26].

### Derivation of oligodendrocyte-containing spheroids

The iPSC-derived oligodendrocytes underwent differentiation following a previously established protocol [27] with some minor modi cations. Briefly, iPSC lines were cultured on vitronectin-coated plates and maintained in Stemflex^™^ medium until they reached 70–90% confluency. These cultures were then dissociated into single cells using ACCUTASE^™^. Once dissociated, the cells were plated on Matrigel^®^-coated six-well plates at a density ranging from 5^10^5^ to 1^10^6^ cells per well in StemFlex^™^ medium supplemented with RevitaCell^™^. The cells were incubated at 37°C with 5% CO_2_ for 1–2 days until colonies with a diameter of approximately 100–250 μm were formed. Upon reaching the desired size, colonies were induced using Neural Induction Media (NIM) supplemented with SB431542 (10 μM), LDN193189 (250 nM), or retinoic acid (100 nM) for 8 days, with daily complete media changes.

On day 8, the cells were transitioned to N2 medium (DMEM/F12 with L-glutamine, N2 supplement (1x), non-essential amino acids (NEAA), Penicillin-Streptomycin (PenStrep), and 2-mercaptoethanol) supplemented with retinoic acid (100 nM) and a smoothened agonist (1 μM). The cells were maintained in N2 medium for 4 days, with daily media changes with the addition of fresh supplements. Overconfluent monolayers exhibiting 3D structures were mechanically disrupted in a lattice-like pattern using a scalpel to generate aggregates. The newly formed aggregates were detached from the wells using a cell lifter and plated onto ultralow attachment plates at a 1:2 ratio with N2B27 medium (N2 medium with B27 supplement lacking vitamin A), supplemented with retinoic acid (100 nM) and a smoothened agonist (1 μM). The cells were cultured in N2B27 medium for 8 days, with 2/3 media replacement every other day.

The cells were subsequently switched to PDGF medium (DMEM/F12 with L-glutamine, N2 supplement (1x), NEAA, PenStrep, 2-mercaptoethanol, PDGFaa (10 ng/ml), IGF-1 (10 ng/ml), HGF (5 ng/ml), NT3 (10 ng/ml), T3 (60 ng/ml), biotin (100 ng/ml), cAMP (1 μM), and insulin 25 μg/ml) with 2/3 media replacement every other day for 10 days. Round aggregates with a diameter ranging from 300 to 800 μm and a golden or brown appearance were selectively harvested and plated onto PLO/Laminin-coated plates at a density of 20 aggregates per well of a 6 well plate, using Glial medium (DMEM/F12 with L-glutamine, N2 supplement, NEAA, PenStrep, 2-mercaptoethanol, HEPES, T3, biotin, cAMP, insulin, ascorbic acid). The aggregates were maintained in the PLO/Laminin plates for 45 days with 2/3 media replacement every other day.

### Nuclei isolation for 10x Chromium Single-cell Multiome ATAC + Gene Expression

On day 76 after the induction of differentiation, the cultures were harvested, and nuclear isolation was accomplished following a 10x protocol: “Nuclei isolation from complex tissues for single-cell multiome ATAC + gene expression sequencing” with minor adjustments. Briefly, the cultures were detached from the plates using ACCUTASE^™^ at 37°C for 5 minutes and then washed with PBS + 0.04% BSA. The cell pellet was then resuspended in RDD buffer with DNAse I (0.1 U/μL) and incubated on ice for 5 minutes. After DNAse I treatment, the cells were washed twice with PBS + 0.04% BSA. The pellets were subsequently resuspended in NP40 lysis buffer, incubated on ice for 5 minutes, and homogenized using a pellet pestle with a cordless motor. The homogenate was then ltered through a 40 μm strainer and pelleted. Nuclei resuspension buffer was added to the pellet, followed by a 5-minute incubation on ice. The pellet was then resuspended and washed twice with nuclei resuspension buffer. Lysis efficiency was assessed by staining with trypan blue and nuclei counted using the Countess^®^ II FL Automated Cell Counter. After verification that lysis reached > 95%, the nuclei were pelleted, resuspended in 0.1X lysis buffer, incubated on ice for 2 minutes, washed once with wash buffer, and resuspended in cold diluted nuclear buffer (1x). The number of nuclei was determined using a Cellometer (Nexcelom). The nuclei were diluted to a concentration of 5,500 nuclei/μL for a target cell recovery of 10,000 nuclei. The diluted nuclei suspensions were used for 10x Chromium Single-cell Multiome ATAC + Gene Expression library preparation according to the manufacturer’s protocol.

### Data analysis

#### RNA-seq

The raw RNA-seq data were preprocessed to remove noise, correct for batch effects, and perform quality control checks. Clustering analysis was performed using Seurat’s graph-based clustering algorithm to identify distinct cell populations. After clustering, similar nuclei with a nal resolution of 0.2 in Seurat were clustered leading to the identification of 11 distinct clusters. Cluster identity was assigned based on the expression of lineage-specific marker genes as follows: 0: Astrocytes; 1: Astrocytes; 2: Excitatory neurons; 3: OPC; 4: Inhibitory neurons; 5: Endothelial; 6: Astrocytes; 7: Cycling progenitors; 8: Oligodendrocytes; 9: Inhibitory neurons; and 10: Excitatory neurons (Supplementary Fig. S1). The differential expression of genes (DEGs) between ancestral groups within each cluster was determined using the MAST test, which employs a generalized linear model framework incorporating cell detection rates within replicates and across groups as a covariate [28]. DEGs were defined as meeting two criteria: p value adjusted < 0.05 and a fold change greater than 1 or less than − 1. Compared with other single-nucleus differential expression methods, the MAST test is known for its low error and false discovery rates [29].

### Pathway analysis

The R library gprofiler [30] was used for functional enrichment analysis. We rst selected the gene symbols of down and up regulated genes using adjusted p-value of < 0.05, and log2FC > 0.32 or < −0.32. The function gost was used to perform the gene set enrichment analysis for each comparison using KEGG pathways database. Multiple comparison correction of enrichment scores was done with the ‘gSCS’ method and pathways with p-adj < 0.05 were considered significant.

#### ATAC-seq

Analysis of the snATACseq data was performed using the ArchR software package v1.0.1 [31] as previously described [14]. Briefly, clustering of cells was performed with ve iterations of latent semantic indexing (LSI) followed by batch correction with Harmony [32]. We created pseudobulk replicates combining all EU, AF, and AI samples separately and called peaks for each ancestry within each cluster via the addReproduciblePeakSet function in ArchR via the default parameters of the MACS2 callpeak command v2.2.7.1 [33]. Differential accessibility for each peak within each cluster was calculated via ArchR getMarkerFeatures.

#### ATAC peak annotation

The function annotatePeak from Chipseeker R library [34] was used to annotate peaks with the nearest gene and genomic region. The annotation was done at the transcript level using the GENCODE V44 database, with the following priority: “Promoter”, “5UTR”, “3UTR”, “Exon”, “Intron”, “Downstream”, “Intergenic”, and the distance of ± 3 kb from the transcription start sites (TSS) was used to assign a peak to a gene promoter-TSS.

#### Hi-C

The in situ Hi-C libraries were constructed following the protocol outlined by Rao et al. [35]. In summary, each library yielded 450–550 million paired-end 150 base pairs reads. Over 270 million nonredundant, uniquely mapped, paired end reads were subsequently utilized for further analysis of each library. Contact matrices were created at resolutions of 500 kb, 50 kb, and 5 kb. The Directionality Index (DI) value of 40 kb bins was employed to identify the topologically associated domains (TADs). To accurately map enhancer‒promoter interactions, the HiCorr pipeline was employed to correct for Hi-C bias at the sub-TAD level and identify Hi-C loops [35].

## RESULTS

### Generation of ancestry-specific iPSCs and iPSC-derived oligodendroglia

**Figure 1:**
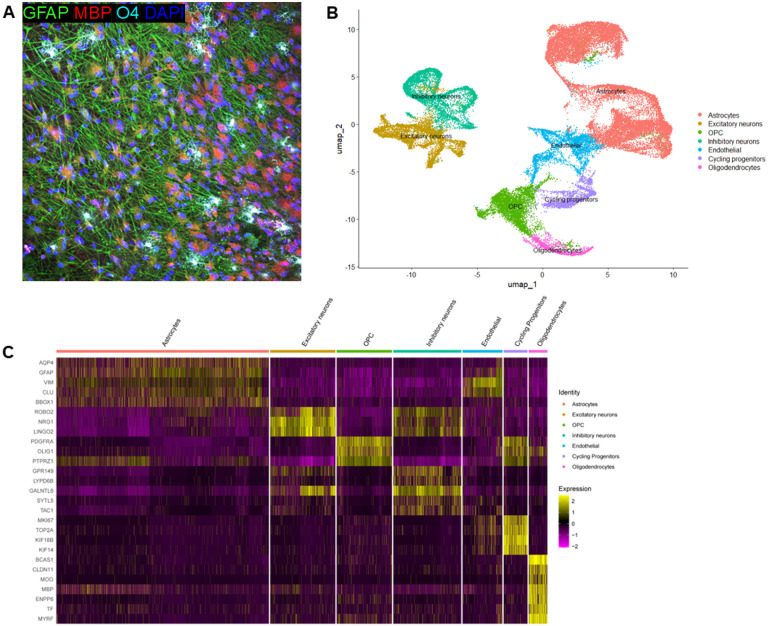
Characterization of iPSC-derived neural spheroids with different ancestral backgrounds. **A.** Representative image of immunocytochemistry analysis of iPSC derived neural 3D cultures. **B.**Visualization of single nuclei clusters from 47,898 nuclei integrated across 12 samples of iPS-derived neural 3D cultures including 4 samples from each ancestry (AF, AI, EU).Clustering of single nuclei from the integrated datasets representing the identified cellular clusters based on scRNA-seq data and grouped by cell type. **C.** Visualization of different cell marker expression in the different cell clusters shown in B. Cells are Expression depicted from purple (low) to yellow (high).

We generated a total of 12 iPSC lines (four African, four Amerindian, and four European) in this study, of which six were from AD patients and six were from non-cognitively impaired individuals (Table 1). In addition, we had four *APOE4/4* carriers and eight *APOE3/3* carriers. We differentiated these 12 lines into neural spheroids producing oligodendrocyte lineage cells at different maturation stages ranging from cycling progenitors through to mature myelinating oligodendrocytes. In addition to oligodendrocyte-related cells, the spheroids contained neurons and astrocytes based on immunocytochemistry (Fig. 1A) and snRNA-seq (Fig. 1B–1C) analyses.

#### Single-nuclei transcriptional profiling of iPSC-derived oligodendroglia containing neural spheroids across diverse ancestries.

Attempts to isolate individual cell types from the tightly clustered spheroids via FACS or pull-down procedures resulted in a low yield of viable cells. To overcome these di culties in recovery and minimize the manipulation of the cells, single-nuclei approaches were used to analyze the transcriptional states of the cells in these oligodendrocyte-enriched neural spheroids. These analyses were performed across the 12 iPSC lines from the three ancestral populations. We characterized a total of 47,898 cells, with an average of 3,991 ± 1,608 per sample. Seurat-based pattern recognition clustering identified seven cell clusters shared by all the individual samples (Fig. 1B and Supplementary Fig. S2). We subsequently detected genes that were enriched in each cluster and utilized these for the assignment of cell types to each individual cluster. We identified one OPC cluster (enriched for the expression of *PDGFRA, COL20A1, OLIG1, OLIG2, and PTPRZ1*) (Fig. 1B–1C) and one oligodendrocyte (OL) cluster (*MOG, CLDN11, ENPP6, MYRF*, and *MBP*) (Fig. 1A–1B) among other cell types, including astrocytes, excitatory and inhibitory neurons, endothelial cells, and cycling progenitors.

### Differentially expressed genes (DEG) between ancestries

**Figure 2:**
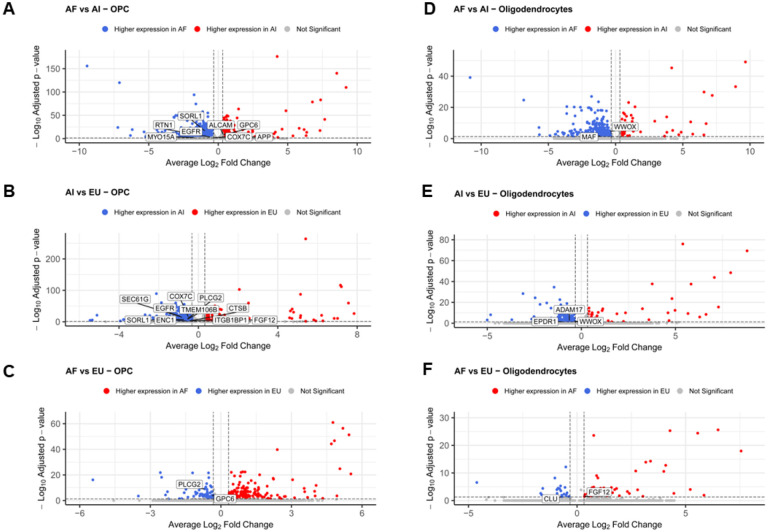
Ancestry-Related Transcriptomic Changes in Oligodendroglia. Volcano plots illustrating differential gene expression analyses for oligodendroglia clusters, specifically Oligodendrocyte Progenitor Cells (OPC) and mature Oligodendrocytes, across diverse ancestry backgrounds. Each plot represents a pairwise comparison of ancestry groups, highlighting the number of differentially expressed genes (DEGs). **A.** European vs. Amerindian comparison in the OPC cluster. **B.** European vs. Amerindian comparison in the Oligodendrocyte. **C.** African vs. Amerindian comparison in the OPC cluster. **D.** African vs. Amerindian comparison in the Oligodendrocyte cluster. **E.** European vs. African comparison in the OPC cluster. **F.** European vs. African comparison in the Oligodendrocyte cluster.

To investigate ancestry-related changes in the transcriptome of the oligodendroglia found in our cultures, we performed differential gene expression analyses using pairwise comparisons of the OPC and oligodendrocyte clusters independently (shown in Fig. 2 and Table 2). In the OPC cluster, we found that the number of DEGs was greatest in the European vs. Amerindian comparisons, with a total of 1,750 DEGs (Fig. 2A, Tables 2 and S1) and approximately 90% (1,573) were increased in the European samples. On the other hand, in the OL cluster, the greatest number of DEGs was found between the African and Amerindian lines, with 492 DEGs, of which ~ 87% (427), were increased in the African lines (Fig. 2D, Tables 2 and S4). To identify DEGs common to the oligodendroglia lineage, we analyzed the data to identify overlapping DEGs in both clusters for each comparison (shown in Table 2).

### Ancestry-dependent changes in AD

**Figure 3:**
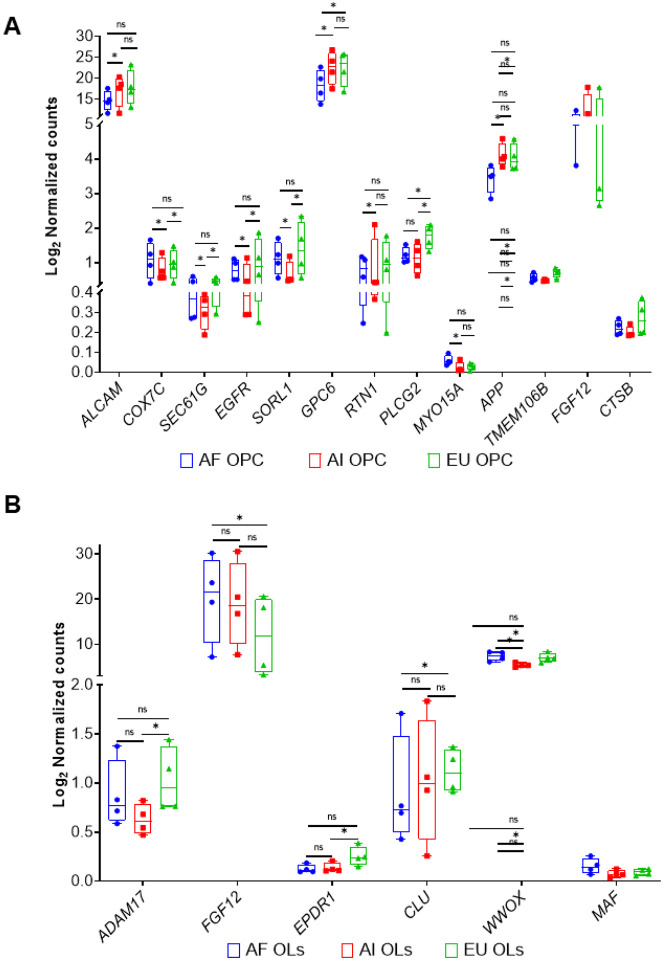
Ancestry-Dependent Expression of Alzheimer’s Disease GWAS Hits and Related Genes in iOPC and iOligodendrocyte Clusters. Boxplots showing gene expression differences between **A.**oligodendrocyte precursor cells (OPC) and **B.** oligodendrocytes (OL) in relation to Alzheimer’s disease (AD) and ancestry-dependent changes.

We compared the DEGs obtained from the different comparisons to genes currently implicated as AD GWAS loci [1, 3, 8, 36, 37]. We found eleven AD GWAS genes (*ALCAM, APP, COX7C, EGFR, GPC6, MYO15A, PLCG2, RTN1, SEC61G, SORL1, and TMEM106B*) to be differentially expressed between the ancestries in the iOPC cluster (Fig. 3A, Table 3). On the other hand, in the iOL cluster, only five AD GWAS hits were differentially expressed: *ADAM17*, *CLU*, *EPDR1*, *FGF12*, *MAF and WWOX* (Fig. 3B, Table 3). One additional interesting DEG was *OPCML*, a gene that has recently been linked to AD [38–40]; in this case, we found it to be increased in Europeans compared with Africans.

### Transcriptome comparisons between APOE 3/3 vs APOE 4/4

**Figure 4:**
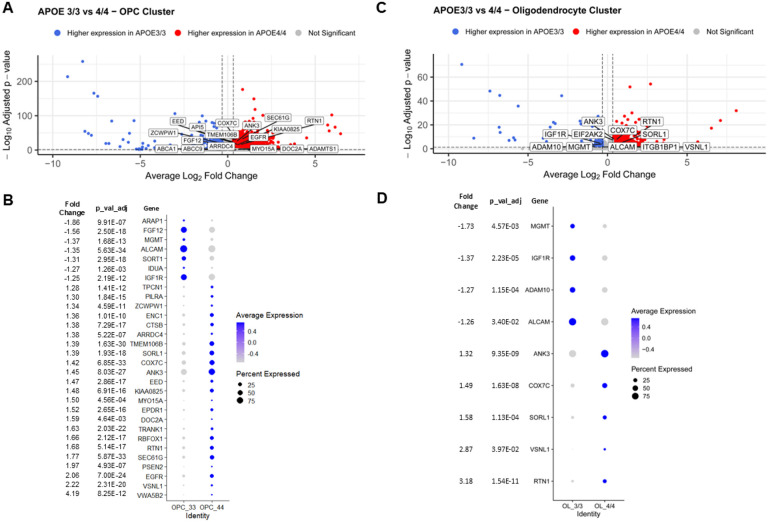
Transcriptional Changes in Oligodendroglia Based on APOE Genotype. Differential gene expression occurring in oligodendroglia based on APOE genotype, comparing the *APOE3/3*lines to the *APOE4/4* lines, irrespective of their ancestry. **A.** Volcano plot showing DEGs between *APOE3/3* and *APOE4/4* samples in the iOPC cluster. Genes that have been reported as AD GWAS hits are labeled. **B.** Dot plot illustrating the pseudo-bulk expression of the differentially expressed AD GWAS hits in the iOPC cluster. **C.** Volcano plot showing DEGs between *APOE3/3*and *APOE4/4* samples in the iOL cluster. Genes that have been reported as AD GWAS hits are labeled. **D.** Dot plot illustrating the pseudo-bulk expression of the differentially expressed AD GWAS hits in the iOL cluster.

To investigate the transcriptional changes that occur in oligodendroglia dependent on the *APOE* genotype, we compared the *APOE3/3* lines with the *APOE4/4* lines irrespective of their ancestry. For the larger iOPC cluster, there were 5,578 DEGs (Fig. 4A, Table S17), 30 of which are known AD GWAS loci; interestingly, most of them were upregulated in the 4/4 lines compared with the 3/3 lines (Fig. 4A,4B), with the exceptions of *IGF1R, IDUA, SORT1, ALCAM, MGMT, FGF12 and ARAP1*. In the iOL cluster, we identified 1,653 DEGs, including 9 AD GWAS genes namely, *RTN1, VSNL1, SORL1, COX7C, ANK3, ALCAM, ADAM10, IGF1R and MGMT* (Fig. 4C, 4D, Table S18). A total of 1,075 DEGs were shared by both clusters, including the *AD GWAS* genes *ALCAM, ANK3, COX7C, IGF1R, MGMT, RTN1, SORL1 and VSNL1* (Tables S17 and 18).

**Figure 5:**
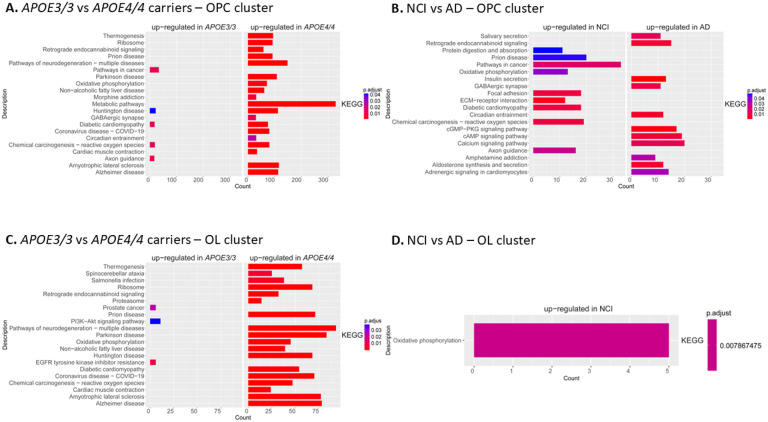
Pathway analysis of DEGs found in oligodendroglia when compared by APOE genotype and AD status **A.**Pathways enriched in OPCs from *APOE4/4* carriers compared to *APOE3/3* carriers. **B.** Pathways enriched in iOLs from *APOE4/4* carriers compared to *APOE3/3*carriers iOPC. **C.** Pathways enriched in iOPCs from AD compared to NCI. **D.** Pathways enriched in iOLs from AD compared to NCI.

**Figure 6:**
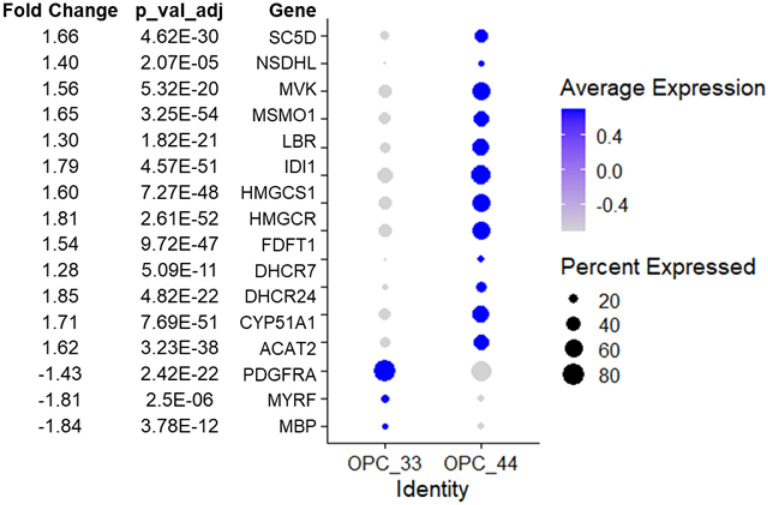
Differential expression of genes implicated in cholesterol biosynthesis and myelination between iPSC derived OPCs with different APOE genotypes. Dot plot illustrates the pseudo-bulk expression of the differentially expressed genes (p_val_adj<0.05 and Fold change >1 or <−1)

To determine whether the overlapping DEGs in oligodendroglia when analyzed by APOE genotype and AD status were involved in specific processes, we performed pathway analysis on these DEGs (Fig. 5). These analyses showed that a wide range of pathways, especially those associated with neurodegenerative diseases, are significantly upregulated in *APOE4/4* carrier iOPCs compared to *APOE3/3* carriers. Many of these pathways are involved in neurodegenerative diseases, including Alzheimer’s disease, Parkinson’s disease, and Huntington’s disease (Fig. 5A). Interestingly, fewer disease-related and more general pathways were identified in iOLs (Fig. 5B). The largest number of genes in iOPCs were involved in metabolic pathways, which led us to test for those involved in cholesterol biosynthesis. Indeed, a large number of DEGS were specifically increased in the cholesterol biosynthesis pathway in *APOE4/4*s compared to *APOE3/3* iOPCs (Fig. 6). Further, we observed reduced expression of *MBP, PDGFRA*, and *MYRF*, key genes in myelin formation in the *APOE4/4* iOPC lines. Finally, Fig. 5C and 5D shows pathway differences observed between NCI and AD iOPCs and iOLs. These pathways were similar to those seen in the APOE 3/3 vs APOE 4/4 comparison likely due to the fact that the majority of AD samples had the *APOE4/4* genotype.

### iOPC and iOLs correlated well with brain transcriptomes

To further validate the identity of our oligodendroglia, we ran Spearman correlation tests to compare our iOPC and OLs iOLs with gene expression patterns from the same cell types in human brain samples [15]. The correlation coeficient between iOPCs and brain OPCs was 0.89 and that between iOLs and brain OLs was 0.85, suggesting that our iOligodendroglia are transcriptionally similar to brain oligodendroglia from aged individuals.

### Males vs Females Expression

To account for sex-driven differences, given that most of our samples were females, except for two of the Amerindian lines, we performed differential expression analyses between the two AI males and the two AI females. It is worth noting that we observed loss of X chromosome inactivation (chromosome X erosion) in the female AI samples (Tables S4 and S8) [41], suggesting that the expression of genes on the X chromosomes and those influenced by them could be affected. Y chromosome genes were expressed as expected (e.g. UTY and USP9Y were expressed in the male derived spheroids but not the female derived). We found that *ALCAM*, *GPC6* and *SORL1* were differentially expressed between males and females in the iOPC cluster (Tables 3 and S4). On the other hand, none of the AD GWAS genes that were differentially expressed between ancestries were differentially expressed between males and females in the iOL cluster (Tables 3 and S8).

### Pathway analysis between ancestries

**Figure 7:**
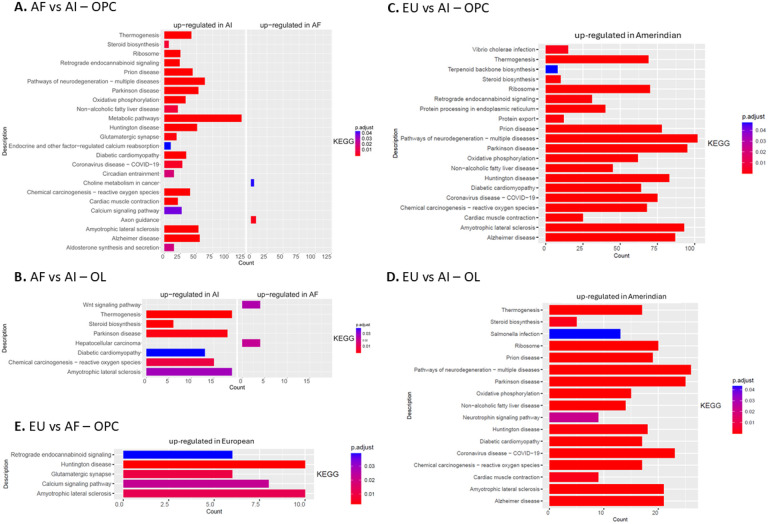
Pathway analysis for DEGs Across Ancestries. DEGs found for each pairwise ancestry comparison in both oligodendroglia clusters were used for pathway analysis. **A.** Pathways enriched between AF and AI iOPCs. **B.** Pathways enriched between AF and AI iOLs. **C.** Pathways enriched between EU and AI iOPCs. **D.** Pathways enriched between EU and AI iOLs. **E.** Pathways enriched between EU and AI OPCs

Pathway analysis between ancestries is shown in Fig. 7. Oxygen species production, diabetic cardiomyopathy, steroid biosynthesis, and several neurodegenerative diseases, including amyotrophic lateral sclerosis (ALS), Parkinson’s disease, and Alzheimer’s disease, were identified in the pathway analyses. Interestingly, pathways of neurodegeneration implicated in multiple diseases, including Alzheimer’s disease, were shown to be enriched for DEGs in Amerindian iOPC and iOL compared with those from European and African ancestries (Fig. 7C and Fig. 7D).

The DEGs whose expression was increased in European oligodendroglia compared with Amerindian oligodendroglia did not appear to converge into any defined pathways. Finally, in the European vs. African comparison, the DEGs enriched in the European samples were also involved in several neurodegenerative disease pathways, specifically Huntington’s disease and ALS, in addition to neuronal signaling processes (Fig. 7E).

#### Single-nucleus chromatin accessibility profiling of iPSC-derived oligodendroglia-enriched neural spheroids across diverse ancestries.

**Figure 8:**
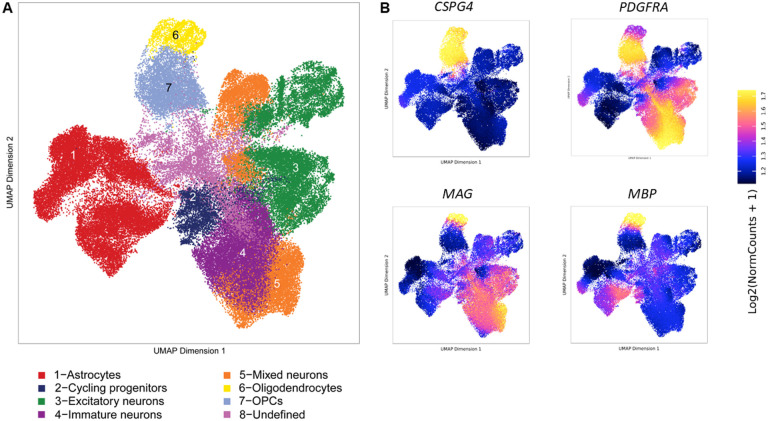
Visualization of single nuclei clusters from 76409 nuclei integrated across 12 samples of iPS-derived neural 3D cultures including 4 samples from each ancestry (AF, AI, EU) **A.** Clustering of single nuclei from the integrated datasets representing the identified cellular clusters based on ATAC-seq data. **B.** Visualization of oligodendroglia markers expression in the different cell clusters shown in A. Accessibility depicted from purple (low) to yellow (high).

To characterize chromatin accessibility pro les associated with different ancestries, we performed single-nuclei ATAC sequencing of oligodendroglia enriched in neural spheroids derived from the 12 iPSC lines. We characterized a total of 76,409 nuclei, with an average of 6,367 ± 3,994 per line. ArchR-based pattern recognition clustering identified 8 cell type clusters shared by all the individual samples (Fig. 8A). Based on marker genes, we identified one OPC (C6) cluster and one oligodendrocyte cluster (C7) (Fig. 8B).

**Figure 9:**
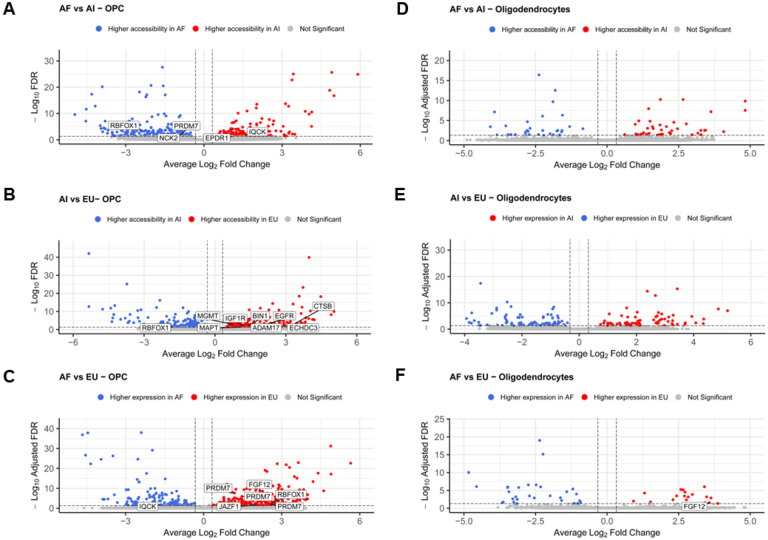
Ancestry-Related Chromatin Accessibility Changes in Oligodendroglia. Volcano plots illustrating differential chromatin accessibility across different ancestral backgrounds in oligodendroglia clusters, specifically iOPC and mature iOligodendrocytes. Each plot represents a pairwise comparison of ancestry groups, highlighting the genes with differentially accessible chromatin. **A.** African vs. Amerindian comparison in the iOPC cluster **B.** European vs. Amerindian comparison in the iOL. **(C)** African vs. European comparison in the iOPC cluster **D.** African vs. Amerindian comparison in the iOL cluster. **E.**European vs. Amerindian comparison in the OPC cluster. **F.** European vs. African comparison in the Oligodendrocyte cluster.

**Figure 10:**
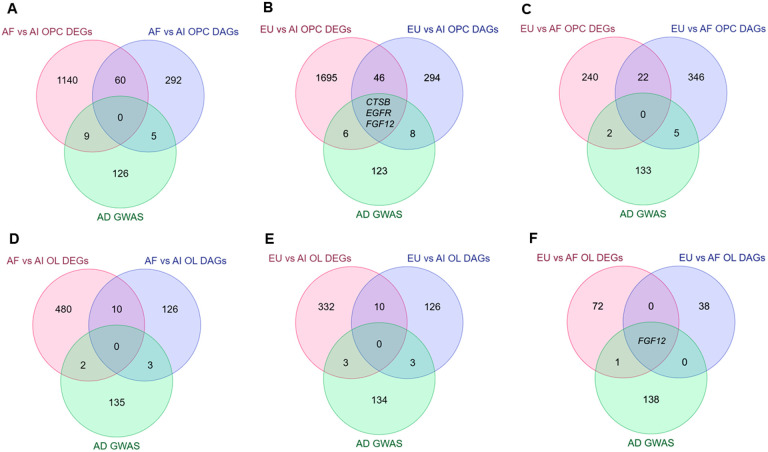
Comparison of Differentially Accessible Genes and Differentially Expressed Genes. Venn Diagram Analysis showing the overlap between differentially accessible genes, differentially expressed genes, and AD GWAS hits for the different pairwise comparisons made based on global ancestry for the OPC (**A, B and C**) and the OL (**D, E and F**) clusters.

To investigate the ancestry-related changes in chromatin accessibility in the oligodendroglia clusters, we performed pairwise comparisons of the iOPC and iOL clusters independently (shown in Fig. 9). For the AF vs. AI comparison in the iOPC cluster, we found 412 differentially peaks (DAPs) distributed across 357 genes, including *RBFOX1, PRDM7, NCK2, EPDR1, and IQCK* (AD GWAS hits) (Fig. 9A, Table S9). The number of DAPs between EU and AI was 413, belonging to 351 different genes, including the *AD GWAS genes ADAM17, BIN1, CTSB, ECHDC3, EGFR, IGF1R, MAPT, MGMT, and RBFOX1* (Fig. 9B, Table S10). We also found DAPs associated with *FGF12* and *PRDM7*, but these same peaks were also found when we compared the two female AI lines to the two male AI lines, limiting our ability to distinguish ancestry-related or sex-related differences in these genes. When comparing the EU to the AF, we found a total of 457 DAPs (Fig. 9C, Table S11). These peaks were distributed across 373 genes, including four *AD GWAS hits: RBFOX1, PRDM7, FGF12, JAZF1, PRDM7, PRDM7, and IQCK*. On the other hand, for the iOL cluster, the greatest number of DAPs was found between AI and EU, with a total of 162 DAPs across 139 genes, including *PDRM7*, *ADAMTS1*, and *REXO1* (Fig. 9E). However, these DAPs were also found in the male vs. female comparison suggesting that the differences seen in accessibility of these genes could be due to sex differences rather than ancestry. We identified 73 DAPs across 63 genes, including a peak in *PDRM7* that was also present in the male vs. female analysis in the comparison between AFs and AIs (Fig. 10E). Finally, we found 49 DAPs across 39 genes between Africans and Europeans, including the AD GWAS risk gene *FGF12* (Fig. 10F).

### Genomic Regulatory Architecture

To examine the relationship between DEG and DAPs, we compared the genes associated with the DAPs with those that showed differential expression for each pairwise comparison in both oligodendroglia clusters – iOPCs and iOLs (Fig. 10). In the iOPC cluster, we identified 60 genes that were differentially accessible and differentially expressed between Amerindians and Africans (Fig. 10A, Table S9). To account for sex-driven differences, we performed differential accessibility analyses comparing the 2 AI males to the 2 AI females (Table S12). Only 17 of the 60 were not found to be differentially expressed/accessible in the comparisons between males and females (Tables S4, S8, S12 and S16); for all of them, increased accessibility was correlated with increased expression. For the AI vs. EU comparison, we found 49 genes that were differentially accessible and differentially expressed; of these, 3 were previously reported AD GWAS genes (*CTSB*, *EGFR* and *FGF12*) [1, 4, 42] (Fig. 10B, Table S10). Among these 49 genes, 23 were not affected by sex and 87% of this accessibility was correlated with expression. The exceptions were *MAP2*, *PPP2R2A* and *PDXDC1*; however, the last gene had two peaks, one in the promoter that correlated with expression and one in an exon that showed an inverse correlation (Table S2 and S10). Finally, in the AF vs. EU comparison, we found 22 DAGs that overlapped with the DEGs we identified (Fig. 10C); in all cases, accessibility correlated with expression, but for one of the genes harboring 2 DAPs, only one located in the promoter region correlated, whereas the other, located at an intron, showed the opposite trend (Table S3 and S11). In the iOL cluster, we found 10 overlapping DAGs and DEGs in the AI versus AF comparison (Fig. 10D, Table S5 and S13), 3 of which were not affected by sex and had direct correlation between accessibility and expression, and 10 in the AI versus EU comparison (Fig. 10E, Table S6 and S14); of these, 7 were not found to be influenced by sex (Table S16), and the accessibility of the peaks was in accordance with their expression.

#### Ancestry-specific differences in *FGF12* chromatin openness and expression

**Figure 11:**
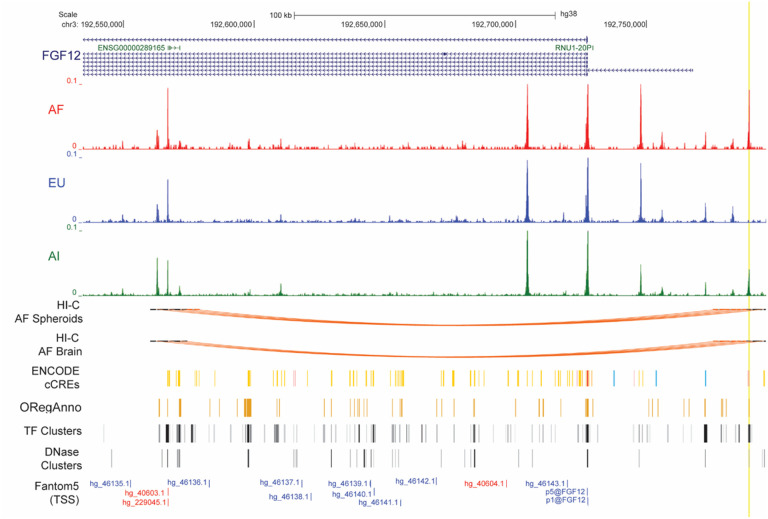
Chromatin accessibility and Hi-C loops overview for a subregion of *FGF12* in oligodendroglia of different ancestries. Highlighted in yellow is a DAP between ancestries with a profile that correlates with *FGF12* expression.

When we looked at the DAP linked to *FGF12* (Fig. 11, yellow highlight, Table S15), we observed that accessibility was correlated with *FGF12* expression (Fig. 3B), as shown by the absence of a open chromatin peak in the EU lines and corresponding reduction in *FGF12* expression in EU compared with the other two ancestries. Interestingly, within this peak, there is a site registered on ENCODE as a candidate cis-regulatory element with a proximal enhancer-like signature – histone modification H3K4me3 (ENCODE Accession: EH38E2269814) (Fig. 11). Additionally, ORegAnno analysis showed this region also contains a transcription factor binding site for CTCF (ORegAnno ID: OREG1365260) (Fig. 11). Further, Hi-C analyses showed chromatin loops occurring between this ATAC peak and the transcriptional start site of the non-coding RNA ENSG00000289165, an antisense to *FGF12*, in spheroids and brain samples (Fig. 11). This finding suggests that transcription of *FGF12* could be modulated in an ancestry-dependent manner by this non-coding RNA.

#### APOE genotype-based comparisons

**Figure 12:**
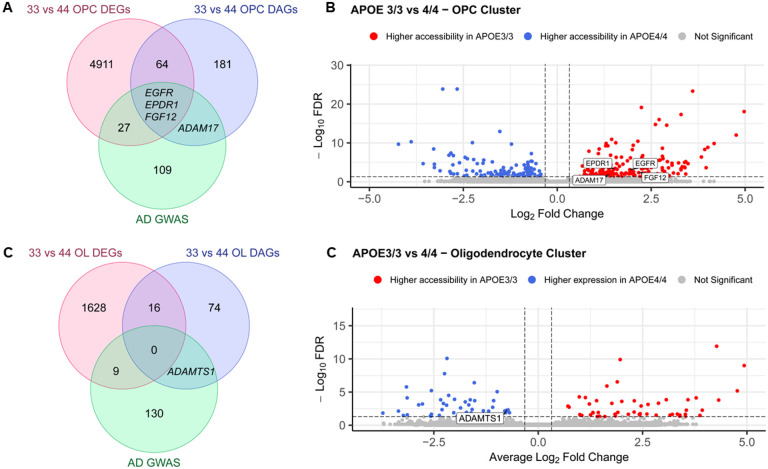
Comparative analysis of differentially expressed genes (DEGs) and genes with differentially accessible peaks (DAPs) in oligodendroglia based on APOE genotype. **A-B.** Comparison of *APOE3/3* OPCs to *APOE4/4* OPCs. **C-D** Comparison of *APOE3/3*Oligodendrocytes to *APOE4/4* Oligodendrocytes.

When comparing *APOE3/3* iOPCs to their *APOE4/4* counterparts, we identified a total of 67 DEGs that had corresponding DAPs. Of these, 3 were AD GWAS genes – EGFR, EPDR1, and *FGF12* (Fig. 12A–B, Tables S17 and S21). Additionally, we identified a DAP associated to *ADAM17* (Fig. 12A–B)., a gene previously reported as an AD GWAS gene thought to be involved in TREM2 processing and regulating APP expression and therefore Aß formation [43]. On the other hand, in the iOL cluster, only 16 DEGs with corresponding DAPs were identified. None of these iOligodendroglia-specific DAPs have, as of yet, been linked to AD (Fig. 12C–D, Tables S17 and S21).

### Disease status-based comparisons

**Figure 13:**
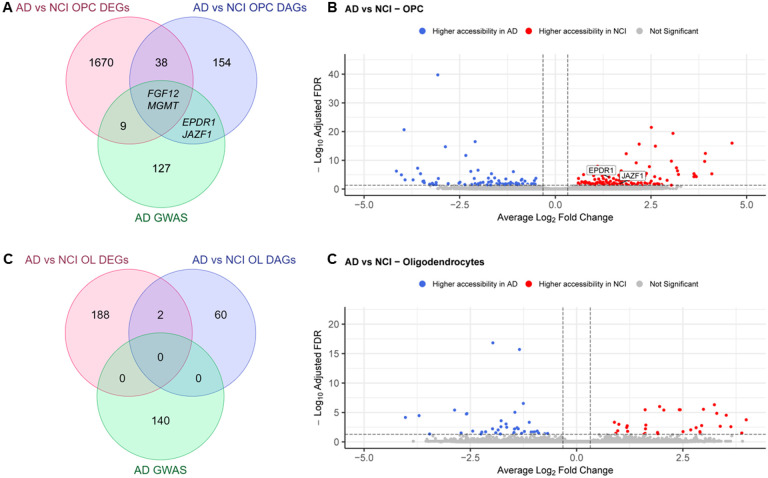
Comparative analysis of differentially expressed genes (DEGs) and genes with differentially accessible peaks (DAPs) in oligodendroglia based on Alzheimer’s disease status. **A-B.** Comparison of OPCs from affected donors to non-cognitively impaired donors OPCs. **C-D** Comparison of Oligodendrocytes from affected donors to Oligodendrocytes from unaffected controls.

When comparing the NCI lines to AD iOligidendroglia, we identified 38 DEGs that had corresponding DAPs, including 2 AD GWAS genes – *FGF12* and MGMT (Fig. 13A–B, Tables S19 and S23). Additionally, we identified DAPs associated with EPDR1 and JAZF1, but these genes were not differentially expressed in this comparison (Fig. 13A–B). Finally, in the OL cluster, we found only 2 DEGs with DAPs, none of which were AD GWAS genes (Fig. 13C–D Tables S20 and S24).

## Discussion

In this study, we compared the chromatin accessibility and transcriptomes of the three different ancestries that contribute to the African American and Hispanic/Latino admixed populations. Most previous studies have focused on the analysis of Europeans. Thus, this study provides new information and iPSC resources on these previously underrepresented ancestries. While we are focused on AD, the data generated can be more broadly applied to other disorder involving OPCs and OLs.

Overall, our studies show that chromatin accessibility and transcriptomes are similar for the three ancestries, although several key known AD genes showed ancestry-specific patterns of expression and/or chromatin openness that may reflect differences in risk between the ancestries. This ancestral similarity was particularly true for the chromatin structure analyses (ATACseq). Our data supports that DEG and DAP for the corresponding genes at AD GWAS loci are not a major factor in most comparisons. This is not to say that the AD (or other disease) GWAS loci that have common or different patterns of expression and chromatin openness will have similar causal mechanisms. We may well find that chromatin conformation analysis (i.e. Hi-C studies) will identify unique differences. Further, while single nuclei approaches have significant power to identify differences between individuals, the overall representation of a population is limited by the number of individuals that can be included in iPSC studies. However, the similarity between ancestries in both accessibility and expression in iOPC and iOLs across the majority of genes is reassuring for future therapeutic efforts in AD.

Both *APOE4/4* and *APOE3/3* carriers across all the ancestries were included in this study. We found that *APOE4/4* carriers across all ancestries and in multiple individuals had clear upregulated expression of genes involved in cholesterol biosynthesis, particularly in iOPCs. The lower numbers of differential genes in iOLs could be due to the relatively fewer numbers of these cells found in this cluster within these cultures. This finding builds upon a similar observation seen in *APOE4/4* oligodendrocytes and OPCs from brain prefrontal cortex in previous studies [44]. Blanchard et al found similar results in a single isogenic APOE 3/3 < > *APOE4/4* iOL line [44]. Thus, cholesterol dysregulation appears to occur early in life during early stages in the development of oligodendrocytes (OPC) leading to impaired cholesterol and lipid biosynthesis. Even mild differences in cholesterol and lipid metabolism by oligodendroglia, occurring over the course of a lifetime, could produce pathogenic results. Indeed, research has highlighted the importance of white matter abnormalities in AD, including those involving oligodendroglia and myelin [18, 19, 45–47]. White matter abnormalities are considered early neuropathological events in AD since white matter hyperintensities, suggesting damage, have been observed preceding the development of AD symptoms [19]. These irregularities in white matter may play a significant role in the pathogenesis of AD and be of considerable diagnostic value [19]. Clinically, there have been reports of early AD-associated anatomical changes in infants and children. For example, Dean et al., reported that in areas affected by AD infants carrying *APOE4* had lower white matter myelin water fractions on magnetic resonance imaging (MRI) compared to non-carriers [48]. Further, Remer et al., found in young *APOE4* carriers a slower rate of myelination in multiple brain regions compared to that of non-carriers [49]. Thus, our *findings* in iOPCs and OPCs have potential implications for future timing of therapy for *APOE4* carriers [50].

We showed that our 3D neural spheroids produce brain-like oligodendroglia with transcriptomes that had high correlation > 85% with human brain oligodendroglia datasets, including OPCs and a population of early myelinating oligodendrocytes characterized by high expression of BCAS1 (Fard et al., 2017). Additionally, we observed that our ancestry-based data replicates *findings* from human brain studies. For example, we found CLU to be differentially expressed between AF and EU iOLs as previously reported by Griswold et al. in AD human brains [15]. These observations suggest that iOPC and iOLs are a good model to study oligodendroglia that effectively reflects phenomena occurring in vivo in human brains.

OPCs, which are central nervous system (CNS) resident stem cells that are spread throughout the brain forming a category of mobile and actively dividing adult precursor cells capable of developing into oligodendrocytes [51]. OPCs play a very important role in the response to myelin damage due to injury or disease, as they are responsible for maintaining the population of oligodendrocytes and participate in myelin repair [52, 53]. In AD, the brain’s microenvironment undergoes detrimental changes that significantly impact OPCs and their functionality [54]. The observed changes in OPCs suggest reduced differentiation capacity and elevated OPC senescence [55]. These changes can have implications for myelin maintenance and repair, as well as for the overall integrity of the central nervous system [54, 55], thereby making OPCs a highly relevant cell population for the study of AD.

In this context, this report is the first study focusing on human iPSC-derived oligodendroglia that includes samples from different ancestries. When we isolated the pathway changes in cholesterol biosynthesis to specific ancestries, we found that several of the cholesterol biosynthesis genes are increased in AI compared to AF and EU Oligodendroglia (Tables S4 and S8). This finding is interesting in light of epidemiological observations in AI populations which suggest that evolutionary pressures favored the increased expression of lipid metabolism genes (including *APOE4*) to overcome food scarcity and endemic infectious diseases (high cholesterol supports innate immunity) in AI groups, particularly in Indigenous groups in South America [56]. This is also believed to be a reason *APOE4* has its highest global frequency in specific regions of South America and Africa [57].

Finally, the inclusion of multiple ancestries in AD research not only supports the importance of reducing health disparities in AD, the comparison of differences between ancestries is a useful research tool in exploring the mechanisms of disease. While the outcome of AD may be similar across ancestral populations, the starting points and biological paths taken in the development of AD may differ between genetic ancestries and environments. Thus, studies on diverse populations provide novel windows into the pathology of AD which will only help move us towards the ultimate goal of prevention.

## CONCLUSIONS

This study represents a significant advancement in understanding the ancestry-specific differences in iPSC-derived oligodendroglia and their implications for Alzheimer’s disease (AD). By generating and analyzing iPSC lines from AF, AI, and EU ancestries, we have identified unique transcriptional and chromatin accessibility profiles in genes that may contribute to AD risk.

Our findings emphasize the importance of including diverse ancestries in iPSC studies to uncover subtle yet crucial differences that may impact disease susceptibility and progression. The differential expression and accessibility of genes associated with AD across ancestries, such as APOE, highlight potential pathways that could be targeted for therapeutic interventions. Notably, the upregulation of cholesterol biosynthesis genes in AI iPSC-derived oligodendroglia suggests evolutionary adaptations that may confer advantages in certain environments. Although these alterations may promote survival in the face of potential food scarcities, they may have come at the cost of increasing risk for AD development.

This study also underscores the role of APOE genotype in modulating gene expression related to lipid metabolism, providing insights into early intervention strategies for *APOE4* carriers. The correlation between our iPSC-derived models and human brain oligodendroglia strengthens the validity of using these models to study neurodegenerative diseases, offering a promising avenue for future research in AD biology and therapeutic development.

Finally, this work not only sheds light on the molecular underpinnings of AD across different genetic backgrounds but also underscores the necessity to address health disparities by ensuring diverse representation in research. Understanding the distinct genetic and environmental pathways leading to AD in various populations will enhance our ability to develop effective, personalized prevention and treatment strategies.

## Figures and Tables

**Table 1:** List iPSC lines used in this study.

HIHG ID	Source^1^	Sex^2^	*APOE* genotype	Global Ancestry	Diagnosis^3^	Age	Global Ancestry^4^			
							AI	EU	AF	EA
201816444	This study	F	4/4	AF	AD	67	0.006	0.050	0.944	0.000
201505672	This study	F	3/3	AF	NCI	72	0.000	0.036	0.964	0.000
202001540	This study	F	3/3	AF	AD	84	0.003	0.082	0.915	0.000
201917729	This study	F	3/3	AF	MCI	90	0.019	0.046	0.935	0.000
202001087	This study	F	4/4	AI	AD	86	1.000	0.000	0.000	0.000
202001079	This study	F	3/3	AI	NCI	71	0.920	0.077	0.000	0.002
201917151	This study	M	3/3	AI	NCI	86	0.955	0.042	0.000	0.003
202001121	This study	M	3/3	AI	NCI	86	0.963	0.011	0.013	0.013
201806025	ADRC	F	4/4	EU	AD	75	0.002	0.995	0.004	0.000
201806023	ADRC	F	3/3	EU	NCI	70	0.007	0.938	0.055	0.000
201806022	ADRC	F	3/3	EU	NCI	65	0.000	0.997	0.003	0.000
201616981	This study	F	4/4	EU	AD	76	0.050	0.886	0.048	0.016

1 ADRC= Alzheimer’s Disease Research Center (UCI)

2 F=Female ; M = Male

3 AD=Alzheimer’s disease ; NCI = Non-Cognitively Impaired ; MCI = Mild cognitive impairment

4 Global ancestry was calculated using admixture software. Refer to Methods for more details.

**Table 2:** Differentially expressed genes (DEGs) across ancestries. Total number of DEGs found in each pairwise comparison between different ancestries.

	TOTAL GENES (AD GWAS)					
	iOPC		iOL		iOPC ∩ iOL	
**EU>AI**	1573(8)	1750	290(3)	345	208(0)	246
**AI>EU**	177(1)		55(0)		38(0)	
**AF>AI**	1007(6)	1209	427(2)	492	250(0)	297
**AI >AF**	202(3)		65(0)		47(0)	
**AF>EU**	181(0)	264	49(1)	74	35(0)	45`
**EU>AF**	83(2)		25(1)		10(0)	

∩ Denotes shared DEGs between the iOPC and iOL clusters

**Table 3:** Differentially expressed AD GWAS genes across ancestries

Comparison	Gene	Fold Change	p_val_adj	DEG between males and females*
AF vs AI OPC	*GPC6*	1.27	8.24E-09	Yes
	*ALCAM*	1.26	1.42E-10	Yes
	*APP*	1.26	1.83E-04	No
	*COX7C*	−1.30	1.66E-02	No
	*SEC61G*	−1.38	1.49E-02	No
	*RTN1*	−1.48	6.21E-04	No
	*EGFR*	−1.82	1.83E-06	No
	*SORL1*	−1.96	2.57E-17	Yes
	*MYO15A*	−3.26	7.87E-03	No
EU vs AI OPC	*FGF12*	1.52	2.77E-06	No
	*CTSB*	−1.39	4.80E-03	Yes
	*COX7C*	−1.43	8.05E-17	No
	*PLCG2*	−1.46	5.08E-11	Yes
	*TMEM106B*	−1.47	1.63E-08	No
	*ENC1*	−1.48	1.45E-02	Yes
	*SORL1*	−1.64	1.94E-08	Yes
	*SEC61G*	−1.76	5.28E-15	No
	*EGFR*	−1.88	3.57E-18	No
EU vs AF OPC	*GPC6*	−1.31	9.75E-05	Not applicable
	*PLCG2*	−1.61	7.95E-10	Not applicable
AF vs AI OL	*WWOX*	−1.34	1.95E-05	No
	*MAF*	−2.20	1.47E-02	No
EU vs AI OL	*WWOX*	−1.33	9.59E-03	No
	*ADAM17*	−1.56	2.40E-03	No
	*EPDR1*	−2.01	4.98E-02	No
EU vs AF OL	*FGF12*	1.27	3.16E-02	Not applicable
	*CLU*	−1.63	3.75E-02	Not applicable

* Found to be differentially expressed between AI male and AI female samples in this study (Tables S4 and S8)

## Data Availability

All data generated and analyzed for this study are included in this published article and its supplementary information files. Additional raw files are available from the corresponding author upon reasonable request.

## Abbreviations

AD: Alzheimer’s disease
MCI: Mild cognitive impairment
NCI: Non cognitively impaired
GWAS: Genome wide association studies
GRA: Genomic regulatory architecture
AF: African
AI: Amerindian
EU: European
EA: East Asian
WGS: Whole Genome Sequencing
iPSC: induced pluripotent stem cells
OPC: Oligodendrocyte precursor cells
OL: Oligodendrocytes
iOPC: iPSC derived OPC
iOL: iPSC derived OL
DEG: Differentially expressed gene
DAP: Differentially accessible peak
DAG: Differentially accessible gene
GA: Global ancestry
